# Association of the Cumulative Dose of Radioactive Iodine Therapy With Overall Survival in Patients With Differentiated Thyroid Cancer and Pulmonary Metastases

**DOI:** 10.3389/fonc.2019.00558

**Published:** 2019-06-28

**Authors:** Jing Yang, Rong Zheng, Meng Liang, Yingying Jia, Lin Lin, Jianhua Geng, Shengzu Chen, Ye-Xiong Li

**Affiliations:** ^1^Department of Nuclear Medicine, National Cancer Center, National Clinical Research Center for Cancer, Cancer Hospital, Chinese Academy of Medical Sciences and Peking Union Medical College, Beijing, China; ^2^Department of Radiation Oncology, National Cancer Center, National Clinical Research Center for Cancer, Cancer Hospital, Chinese Academy of Medical Sciences and Peking Union Medical College, Beijing, China

**Keywords:** differentiated thyroid cancer, pulmonary metastases, radioiodine therapy, cumulative dose, patient age

## Abstract

**Purpose:** The optimal cumulative dose of radioactive iodine therapy (RAIT) for patients with differentiated thyroid cancer (DTC) and pulmonary metastases (PM) is not known, therefore we evaluated the association between the cumulative dose of RAIT and overall survival (OS).

**Methods:** A total of 202 patients with DTC and PM who underwent thyroidectomy and RAIT were analyzed in this study. The median cumulative dose of RAIT was 530 mCi. OS was compared with an age- and sex-matched general population from China to assess relative survival. Multivariable proportional hazards model smoothing by penalized spline was applied to identify independent predictors and examine the adjusted non-linear association of cumulative dose of RAIT and patient age with mortality.

**Results:** The observed survival and relative survival at 10 years was 54.96 and 60.81%, respectively, with the standardized mortality ratio being 5.34. The cumulative dose of RAIT was associated with mortality in a dose-dependent fashion without an apparent cutoff point after adjustment of other variables. A linear but moderate association was found in the dose of 300 to 1,000 mCi. Cumulative dose of RAIT, patient age, diameter of pulmonary metastases, and extrapulmonary metastases were identified as independent predictors for OS. The increasing patient age was associated with mortality in a non-linear pattern, with the optimal threshold being 40 years. With advancing age, the risk of death increases rapidly in patients aged 40 years and younger, but slowly in patients over 40 years.

**Conclusions:** RAIT should be assigned to RAI-avid patients until disease has been controlled or RAIT becomes refractory after consideration of the potential long-term side-effects. Patient age was associated with OS in a non-linear pattern, with a threshold at 40 years. Consideration of age as a binary variable could elucidate a more accurate prognosis in such patients.

## Introduction

The incidence of thyroid cancer (TC) has increased persistently worldwide in recent years (1). In China, annual newly diagnosed cases of TC has increased from 54,175 in 2010 to 201,000 in 2015 (2, 3), of which differentiated thyroid cancer (DTC) accounts for >90%. Five-year survival for TC is higher than that for all other cancer sites, and reached 84.3% in 2012–2015 in China (4). However, patients with distant metastases carry an increased risk of death from TC (5). The lungs are the most common organ of distant metastases, accounting for ~70% of all cases of distant metastases (6). DTC patients with pulmonary metastases (PM) have a relatively poor prognosis, with median survival <10 years (7).

For patients with DTC and PM, consensus guidelines recommend that radioactive iodine therapy (RAIT) should be introduced after a total or near-total thyroidectomy to reduce the risk of disease recurrence or mortality (8, 9). Also, RAIT should be first-line therapy for DTC patients as long as the inoperable PM are RAI-avid (10). RAIT courses in patients with DTC and PM are generally based on responses to RAIT. The following course of RAIT would be delivered to patients who concentrate RAI and respond clinically within PM (6, 11). With regard to the cumulative RAI dose, limits at 600 or 1,000 mCi have been recommended in several studies, but they were empiric settings without adjustment for other variables (12, 13). Studies have deemed that patients with RAI-avid PM could obtain survival benefit from RAIT (14). However, whether increasing amount of RAIT translates into long-term survival benefit for such patients is not known (15).

In patients with DTC and PM, patient age is the most important prognostic factor (16). Moreover, it has been reported that age has an association with the response to RAIT (16, 17).Therefore, taking age into account while evaluating the effect of the cumulative dose of RAIT on overall survival (OS) is crucial. Most studies have incorporated age into binary variables with a distinct cutoff point for DTC patients (18). In 2018, the 8th edition of the *Staging System for Primary Tumors* set by the American Joint Committee on Cancer adjusted the age cutoff point from 45 to 55 years, raising concerns among pathologists and clinicians (19–21). Recent studies using age as a continuous variable rather than a dichotomic factor found better concordance with survival and death from TC (22, 23). For patients with DTC and PM, studies have demonstrated that an age of ≥45 years carries a worse prognosis than in patients aged <45 years (24, 25). However, no study has examined the dose-dependent effect of age among patients with DTC and PM.

In this patient cohort with long-term follow-up, we aim to ascertain if there is a dose-dependent effect for RAIT and survival benefits in patients with DTC and PM. Furthermore, we try to examine if there is an optimal dose of RAIT and an age cutoff for survival in patients with DTC and PM.

## Materials and Methods

### Patient Population

We identified cases with a diagnosis of DTC and PM in the National Cancer Center of China, and restricted patient age at diagnosis of PM to older than 18 years. All medical records of DTC patients who had received RAIT in the Department of the Nuclear Medicine of National Cancer Center during this period were reviewed systematically. For RAI-avid patients, PM diagnosis was primarily based on an increased serum level of thyroglobulin (Tg) and positive results in therapeutic iodine 131 (I-131) whole-body scan (WBS), taking pulmonary nodules on radiography or computed tomography (CT) of the chest into consideration. “WBS-positive” was defined by status of I-131 uptake in the lungs higher than the normal basal level (excluding physiologic uptake and contamination from the body surface). With regards to non-RAI-avid patients whose pulmonary nodules were WBS-negative throughout the entire treatment, PM were confirmed by radiography or CT of the chest and increased serum level of Tg during follow-up.

### Treatment

All patients underwent thyroidectomy. When necessary, more than one procedure would be performed on patients to ensure total or near-total thyroidectomy. Central lymph node compartment (level VI lymph nodes) dissection was performed routinely except for cN0 patients with micropapillary carcinoma or well-differentiated follicular carcinoma. Therapeutic lymph node neck dissection would be performed if a suspicious lymph node was detected by pre-operative imaging or physical examination or intraoperative exploration, while prophylactic lymph node dissection was not actively performed. In order to ensure the concentration of radioactive iodine in pulmonary metastases, all patients received surgical treatment before RAI therapy and reoperation would be delivered to the patients who confirmed with cervical lymph node metastasis during RAI therapy. After thyroidectomy, conventional measurements including free tri-iodothyronine, iodothyronine, free thyroxine, thyroxine, thyroid-stimulating hormone (TSH), Tg, ultrasonography of the neck, and CT/radiography of the chest were done before the first course of RAIT. Then, patients were instructed to follow a low-iodine diet for ≥2 weeks. During the 3–4 weeks before the initiation of RAIT, patients were not allowed to take any iodine or iodine-containing substance in order to guarantee the withdrawal of thyroid hormones to achieve an adequate hypothyroid state (serum level of TSH >30.00 uIU/mL).

The radioactive iodine therapy was carried out using fixed-dose method taking into account the physical status of patients. All patients were administered thyroid-hormone therapy after RAIT for 24–48 h. ^131^I-WBS was carried out 3–8 days after oral RAIT to observe RAI absorption in PM, the results of which, combined with a change in serum levels of Tg and tumor sizes on anatomic imaging, were considered to be prognostic indicators for the assessment of PM response to RAIT. After a course of RAIT, patients with smaller or less metastatic pulmonary nodules in imaging examinations, reduced concentration of iodine within PM in I-131-WBS, and reduced Tg levels (compared with inhibition or stimulation of TSH) than that before this administration were thought to be effective. Clinicians made subsequent treatment plans based on the therapeutic response of last course of RAIT. The RAIT would stop once a patient was diagnosed as non-RAI-avid PM, and generally received a referral for radiotherapy or chemotherapy.

### Endpoint and Statistical Analyses

All patients were observed at follow-up even if they were transferred to other hospitals or departments. Patients who completed the entire treatment in our hospital were followed up annually for the first 5 years and then every 2 or 3 years. Patients transferred to other hospitals were followed up by correspondence or telephone contact. Statistical analyses were undertaken on follow-up data collected up to September 2017 or at the time of death from any cause. The primary endpoint was OS (defined from the time of the initial diagnosis of PM to the time of death from any cause or to the final follow-up assessment).

The overall survival was compared with data from an age- and sex-matched population to evaluate the net survival (relative survival) of this cohort of patients with DTC and PM. The Kaplan–Meier method was used to estimate observed survival. Expected OS accounting for age and sex was generated in the R program (Vienna, Austria) using the general population of China as a reference group. Observed survival vs. expected OS was plotted using a conditional approach and expressed as a standardized mortality ratio (SMR) of observed deaths to expected deaths. Then, relative survival was estimated by the Ederer II method to account for competing deaths from other causes. Cox proportional hazards regression with a stepwise procedure was used to identify the independent predictors for OS. The penalized spline (P-spline) fitting the Cox model allowed a non-linear relationship of cumulative dose of RAIT and age with the logarithm (ln hazard ratio [LnHR]) of mortality estimated from the full Cox regression model adjusted for other independent covariates.

## Results

### Patient Characteristics

A total of 202 patients with DTC and PM formed the study cohort. The median age was 50.5 (range, 18–86) years and 57% were female (Table 1). Most patients had: papillary thyroid carcinoma (73%); lymph-node involvement (76%); no extrapulmonary metastases (81%); iodine concentration within PM (74%); not undergone radiotherapy or chemotherapy (79%). All 202 patients underwent thyroidectomy and 167 patients received central cervical lymph node dissection. Only central lymph node dissection was delivered to 52 patients, while additional lateral lymph node dissection was delivered to 115 patients (43 patients received bilateral lymph node dissection and 72 patients received unilateral lymph node dissection). All patients received at least one course of RAIT after thyroidectomy, with the median cumulative dose of RAIT being 530 (range, 1,000–1,650) mCi. Twenty-seven patients developed local recurrence during follow-up, of which, 22 patients had nodal recurrence, and 5 patients had recurrence in the thyroid bed or tracheal wall. Second primary malignancies were identified in six patients after RAI therapy (four cases of lung cancer, two cases of breast cancer). Two patients were diagnosed as having pulmonary fibrosis. Information about other clinical characteristics of patients are listed in Table 1.

**Table 1:** Demographic, clinical, and treatment characteristics of patients with differentiated thyroid cancer and pulmonary metastases (*N* = 202).

**Characteristic**	**No. (%)**
**Sex**	
Male	87 (43)
Female	115 (57)
**Age**	
Range	18–86
Median (IQR)	50.5 (36, 62)
Mean (SD)	49.04 ± 15.74
**Pathology**	
PTC	148 (73)
FTC	47 (23)
FV PTC	7 (4)
**Extrathyroidal extension**	
Yes	72 (36)
No	7 (3)
Unknown	123 (61)
**Lymph-node involvement**	
Yes	153 (76)
No	40 (20)
Unknown	9 (4)
**Diameter of pulmonary metastases (cm)**	
≥1	65 (32)
<1	89 (44)
Unknown	48 (24)
**Extrapulmonary metastases**	
Yes	39 (19)
No	163 (81)
**Extent of surgery (initial procedure)**	
Total thyroidectomy	98 (49)
Thyroid lobectomy	99 (49)
Unknown	5 (2)
**Iodine concentration within pulmonary metastases**	
Yes	149 (74)
No	53 (26)
**Radiotherapy or chemotherapy**	
Yes	42 (21)
No	160 (79)
**Frequency of rait**	
Range	1–10
Median (IQR)	3.5 (2, 5)
Mean (SD)	4.03 ± 2.19
**Cumulative dose (mCi)**	
Range	100–1650
Median (IQR)	530 (300, 850)
Mean (SD)	598.03 ± 349.05

*IQR, interquartile range; SD, standard deviation; PTC, papillary thyroid carcinoma; FTC, follicular thyroid carcinoma; FV PTC, follicular variant of papillary thyroid carcinoma; RAIT, radioactive iodine therapy*.

### Comparison of Survival of the Study Group With That of the General Population

**Figure 1:**
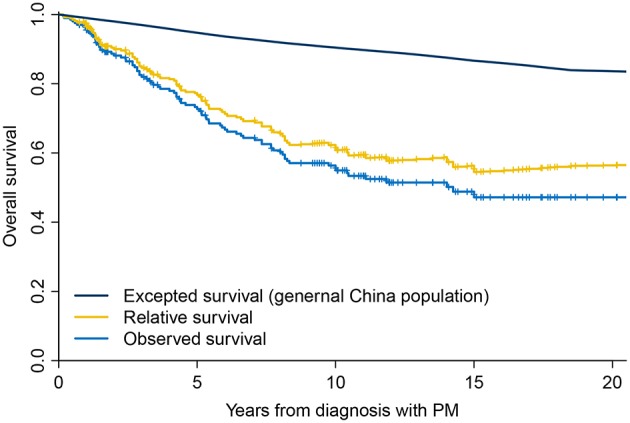
Overall survival of patients with differentiated thyroid cancer and pulmonary metastases compared with the general population of China.

Median follow-up was 93 (range, 2–258) months. There were 86 deaths during the entire follow-up period. The observed OS was 72.68% at 5 years and 54.96% at 10 years (Figure 1). Using the general population of China as a reference, the expected OS was 94.73% at 5 years and 90.43% at 10 years, resulting in relative survival of 76.74% at 5 years and 60.81% at 10 years (Figure 1). Patients with DTC and PM had significantly decreased survival compared with the age- and sex-matched general population of China, with an overall SMR of 5.34 (95% confidence interval (CI), 4.32–6.60; *P* < 0.001).

### Dose-Dependent Effect of RAIT on OS

**Figure 2:**
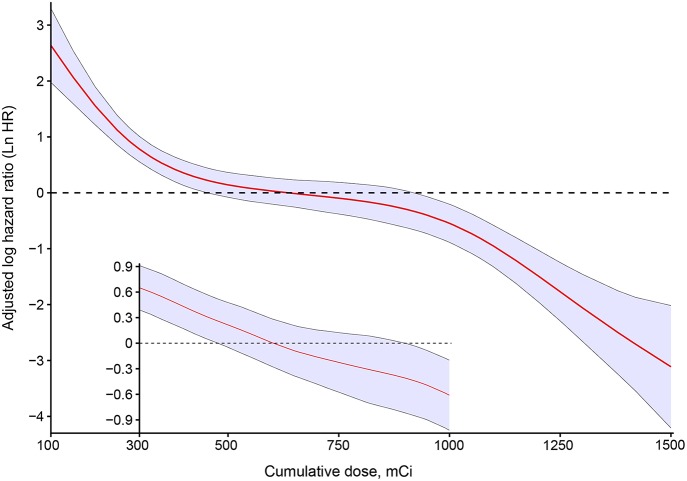
Estimated logarithm hazard ratios with 95% confidence intervals for the association of the cumulative dose of radioactive iodine therapy and overall survival in patients with differentiated thyroid cancer and pulmonary metastases. Adjusted by age, sex, pathology type, extrathyroidal extension, lymph-node involvement, extrapulmonary metastases, diameter of pulmonary metastases, and radiotherapy or chemotherapy. The main plot indicates the association between the cumulative dose of radioactive iodine therapy and overall survival for all patients with RAI-avid PM. The subset of the plot indicates the association among the cases who received between 300 and 1000 mCi.

In an unadjusted analysis, estimates of observed OS and relative survival for 202 patients were positively associated with an increasing cumulative dose of RAIT (Table 2). Survival for patients with a cumulative dose ≤400 mCi (quartile 1) was lowest, whereas survival for patients with a cumulative dose >900 mCi (quartile 4) was highest. Survival was relatively similar in patients with a cumulative dose of RAIT in quartile 2 and quartile 3. To quantify the dose-dependent effect, we entered the cumulative dose of 149 RAI-avid patients as a continuous variable into the multivariate Cox regression using P-splines to allow for non-linear relationships between the RAIT dose and outcomes. This model confirmed that the risk (adjusted LnHR) of death decreased with an increasing cumulative dose of RAIT, and that the risk was expected to decrease moderately from 300 to 1,000 mCi (Figure 2). With respect to the 53 non-RAI-avid patients, mortality decreased with an increasing cumulative dose up to 400 mCi and then plateaued at 500 mCi (Supplementary Figure 1).

**Table 2:** Ten-year estimates for observed survival and relative survival according to the cumulative dose of radioactive iodine therapy.

**Quartile**	**Cumulative dose (mCi)**	***N***	**Events**	**Observed survival, %, 95% CI**	**Relative survival, %, 95% CI**
1	100.00–400.00	55	33	37.84 (25.49–56.18)	41.37 (28.14–60.83)
2	400.01–550.00	47	20	66.27 (49.53–88.67)	71.45 (53.83–94.84)
3	550.01–900.00	53	21	65.59 (51.56–83.43)	74.39 (58.74–94.22)
4	900.01–1650.00	47	12	80.48 (67.60–95.81)	86.56 (73.01–98.62)

Next, we compared the statistical fitness of different multivariate survival models (adjusted by age, sex, pathology type, extrathyroidal extension, lymph-node involvement, extrapulmonary metastases, diameter of pulmonary metastases, radiotherapy, or chemotherapy) with the cumulative dose of RAIT treated as a continuous variable (P-spline or linear) or a binary variable (with cutoff points from 200 to 1,000 mCi) or tertile variable (with cutoff points from 200 to 1,000 mCi). The model that included the cumulative dose of RAIT as a binary variable with a cutoff point at 750 mCi (Akaike Information Criterion (AIC) = 422.59), and the tertile variable with a cutoff point of 300 and 1,000 mCi (AIC = 416.43) had the lowest AIC estimate compared with other models (Supplementary Figure 2). This finding suggested that treating the cumulative dose of RAIT as a tertile variable provided a better data fit. We further assessed the specific dose-dependent pattern for patients with a cumulative dose between 250 and 1,000 mCi, because only five events were observed below or above this range, respectively. As a result, a linear association without an apparent cutoff point between the cumulative dose of RAIT and OS was obtained, thereby reflecting a stable and more accurate dose-dependent relationship (Figure 2).

### Independent Predictors of OS

**Figure 3:**
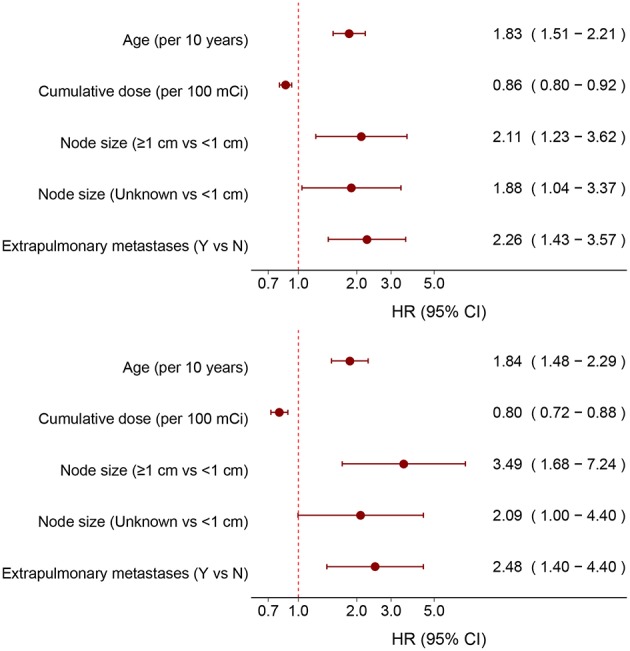
Adjusted hazard ratios of independent predictors for all patients and RAI-avid patients.

After stepwise selection of models, the cumulative dose of RAIT, patient age, diameter of pulmonary metastases, and status of extrapulmonary metastases were placed in the best-fitness model. To assess the magnitude of these effects, we included the cumulative dose of RAIT and age as continuous variables for all 202 patients and 149 RAI-avid patients, respectively. The corresponding adjusted HR was 0.86 (95%CI, 0.82–0.92) and 0.80 (0.72–0.88) per use of 100 mCi on the cumulative dose of RAIT, and 1.83 (1.51–2.21) and 1.84 (1.48–2.29) per 10 year increase in patient age (Figure 3). The risk of death increased for patients with larger diameter of pulmonary metastases and extrapulmonary metastases (Figure 3).

### Non-linear Association Between Age and OS

**Figure 4:**
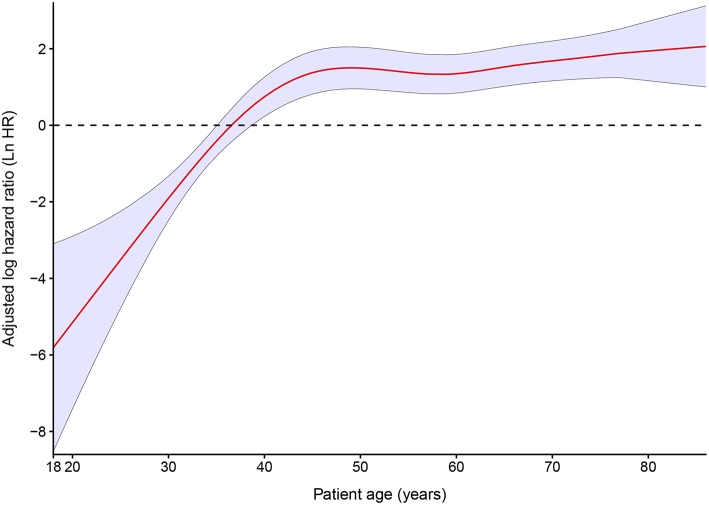
Estimated logarithm hazard ratios with 95% confidence intervals for the association of patient age and overall survival in patients with differentiated thyroid cancer and pulmonary metastases. Adjusted by the cumulative dose of radioactive iodine therapy, sex, pathology type, extrathyroidal extension, lymph-node involvement, extrapulmonary metastases, diameter of pulmonary metastases, and radiotherapy or chemotherapy.

In an unadjusted analysis, 10 year estimates for observed OS for all 202 patients were inversely associated with increasing age, and the 10 year relative OS confirmed this pattern (Table 3). After adjustment for the cumulative dose of RAIT, sex, pathology type, extrathyroidal extension, lymph-node involvement, extrapulmonary metastases, diameter of pulmonary metastases, and radiotherapy or chemotherapy, the P-spline plot demonstrated a significant non-linear association between age and OS in 149 RAI-avid patients, with an apparent cutoff point at ~40 years (Figure 4). A non-linear association between the predictor and outcome can indicate the existence of a cutoff point in this relationship. Next, we compared the statistical fitness of different age–survival models with age treated as a binary variable (with cutoff points at 18 to 86 years). The model that included age as a binary variable with a cutoff point at 40 years had the lowest AIC (408.74) compared with models that cutoff age at other points (Supplementary Figure 3). This finding suggested that patients aged >40 years had an increased risk of death (Supplementary Figure 4).

**Table 3:** Ten-year estimates for observed survival and relative survival according to age.

**Age group (years)**	***N***	**Events**	**Observed survival, %, 95% CI**	**Relative survival, %, 95% CI**
18–29	27	0	100	100
30–39	39	3	94.20 (86.71–100)	94.61 (87.19–100)
40–49	30	15	54.33 (35.24–83.77)	55.74 (36.80–84.43)
50–59	38	19	37.17 (20.46–67.52)	40.34 (22.90–71.08)
60–69	51	35	34.30 (20.29–57.98)	37.33 (20.97–66.45)
70–86	17	14	17.86 (5.35–59.64)	31.17 (10.93–88.91)

## Discussion

For patients with DTC and PM, the cumulative dose of RAIT was associated with mortality in a dose-dependent fashion after adjustment for independent prognostic variables. The risk of mortality decreased with an increase in the cumulative dose of RAIT without an apparent cutoff point. A linear but moderate pattern was found in the dose range 300 to 1,000 mCi. Patient age, diameter of pulmonary metastases, extrapulmonary metastases, and the cumulative dose of RAIT were independent predictors for OS. Age was associated significantly with mortality in a non-linear pattern after adjustment for clinical and treatment characteristics. The optimal threshold for age was 40 years, identifying a less rapid increase in the probability of death with advancing age.

The cumulative dose of RAIT was an independent predictor of OS for patients with DTC and RAI-avid PM. A significant association between an increased cumulative dose of RAIT and survival benefit was observed, a finding that is in accordance with other studies (12, 13, 26, 27). Furthermore, we demonstrated that RAIT prolonged survival in a dose-dependent fashion, allowing for linear relationships between the cumulative dose of RAIT and OS. Although the declining trend was moderate from 300 to 1,000 mCi, survival outcomes were improved persistently with increased cumulative dose. This pattern may have been a result of a progressive reduction of uptake in the lungs, effective half-life, and the radiation dose delivered to the lungs with successive therapies (28–31). Then, we took patients with a cumulative dose between 300 and 1,000 mCi as a subset group and discovered a linear association without an apparent cutoff point between the cumulative dose of RAIT and OS. These results suggested that an increasing dose had a dose-dependent benefit on survival for patients administered RAIT between 300 and 1,000 mCi. The subset analysis may have been more precise because more cases had a dose between 300 and 1,000 mCi. Even though the first two RAIT courses could reduce the mortality risk remarkably, the risk of death for these patients remained high. The effect of the first two courses of RAIT was indicated by Handkiewicz-Junak et al., who found that the recurrence risk was decreased significantly among patients with DTC and distant metastases who received a mean cumulative dose of 300 mCi of RAIT (32). Patients would not receive further RAIT once PM had been diagnosed as refractory. Thus, patients who received >1,000 mCi of RAIT were usually RAI-avid consistently during treatment, which resulted in the best prognosis among all patients. Capdevila et al. stated that RAIT can become refractory at a cumulative dose of 600 mCi (33), whereas other scholars had challenged the suggestion that 600 mCi was a threshold to define RAI resistance (13, 34). An optimal dose for patients with DTC and PM within the range 300 to 1,000 mCi was not found in our study. Our findings suggest that RAIT should be repeated until disease has been controlled or RAIT becomes refractory after consideration of long-term risks. Furthermore, results from 53 non-RAI-avid patients suggested that patients with non-RAI-avid disease usually could not obtain survival benefit from additional RAITs.

In our study, apart from the cumulative dose of RAIT, multivariate analyses suggested that age, diameter of pulmonary metastases, and extrapulmonary metastases status were also independent prognostic factors. A poor prognosis in elder patients with DTC is regarded as common sense by pathologists and clinicians, and recent studies have suggested that age should be treated as a spectrum rather than a specific cutoff point (35). For patients with DTC and PM, the optimal cutoff points for age varied from 40 to 60 years (17, 36–38), but no study has taken age as a continuous variable. Our research suggested that the risk of death for patients with DTC and PM increased sharply along with increasing age until 40 years of age, whereas the trend was moderate for patients aged >40 years. This non-linear pattern supported studies that had taken 40 years as a cutoff point for prognosis prediction (7, 38). The poor prognosis in patients aged >40 years could be attributed to a relatively longer disease duration, higher prevalence of advanced disease, poor curative effect of iodine treatment, a decline in the immune system, and a more aggressive variant of TC (16, 39). According to our study, consideration of age as a continuous variable or advancement of the cutoff point of age to 55 years for patients with DTC and PM was not applicable, and more evidence is needed to elucidate a more accurate staging system.

Although the lungs are the most common metastatic sites of DTC, population-based data suggest that patients with DTC and distant metastases account for <1% of all DTC cases (40). Given the relatively low incidence of patients with DTC and PM, our study provided a relatively large sample size with long follow-up to assess the association between the cumulative dose of RAIT and survival. Our data could be useful for clinicians to decide if they should continue RAI administration in patients with RAI-avid metastatic lung disease despite achieving a cumulative activity >600 mCi. A cutoff point was not found for RAIT, so our study suggests that RAIT should be assigned to RAI-avid patients until disease has been controlled or RAIT becomes refractory after consideration of the long-term side-effects of RAIT. Our data would be helpful to guide RAIT plans for patients with DTC and PM, and might be useful for clinicians to continue the drive toward more individualized management.

Our study had three main limitations. First, this was a retrospective study in a single center in China. A multicenter, international-origin dataset is needed to validate our results. Second, our results were derived from the outcome of all-cause deaths. To address this limitation, we used the general population of China as a reference to calculate relative survival from DTC. Third, some patients were RAI-avid initially but non-RAI-avid after several courses of RAITs. We classified these patients into the RAI-avid group according to the initial response, but the underlying variations may have perturbed the dose-dependent patterns.

## Conclusions

In patients with DTC and PM, OS estimates were positively associated with an increasing cumulative dose of RAIT, and negatively associated with an increasing age. The cumulative dose of RAIT was associated with mortality in a linear dose-dependent fashion, with a downward trend being moderate (but more accurate) in the dose of 300 to 1,000 mCi. The increasing patient age was associated with mortality in a non-linear fashion, with the optimal threshold being 40 years. With advancing age, the risk of death increases rapidly in patients aged 40 years and younger, but slowly in patients over 40 years.

## Data Availability

The datasets generated and/or analyzed during the current study are not publicly available due to data security but are available from the corresponding author on reasonable request.

## Ethics Statement

This study was carried out in accordance with the Declaration of Helsinki and was approved by the Independent Ethics Committee of Cancer Hospital, Chinese Academy of Medical Sciences. Waiver of informed consent was applied to this study because it is a retrospective analysis of existing data.

## Author Contributions

RZ and Y-XL: conception and design and critical revision of the article for important intellectual content. JY: analysis and interpretation of the data and drafting of the article. JY, ML, YJ, LL, JG, and SC: collection of provisions of data. All authors: final approval of the article.

### Conflict of Interest Statement

The authors declare that the research was conducted in the absence of any commercial or financial relationships that could be construed as a potential conflict of interest.
