# Supplementary material for: Association of the Cumulative Dose of Radioactive Iodine Therapy With Overall Survival in Patients With Differentiated Thyroid Cancer and Pulmonary Metastases
Source: Front Oncol. 2019 Jun 28;9:558. doi: 10.3389/fonc.2019.00558 (PMC6609903; doi:10.3389/fonc.2019.00558)
Supplement: Supplementary file 1 [file Data_Sheet_1.docx]

**Supplementary Figures**


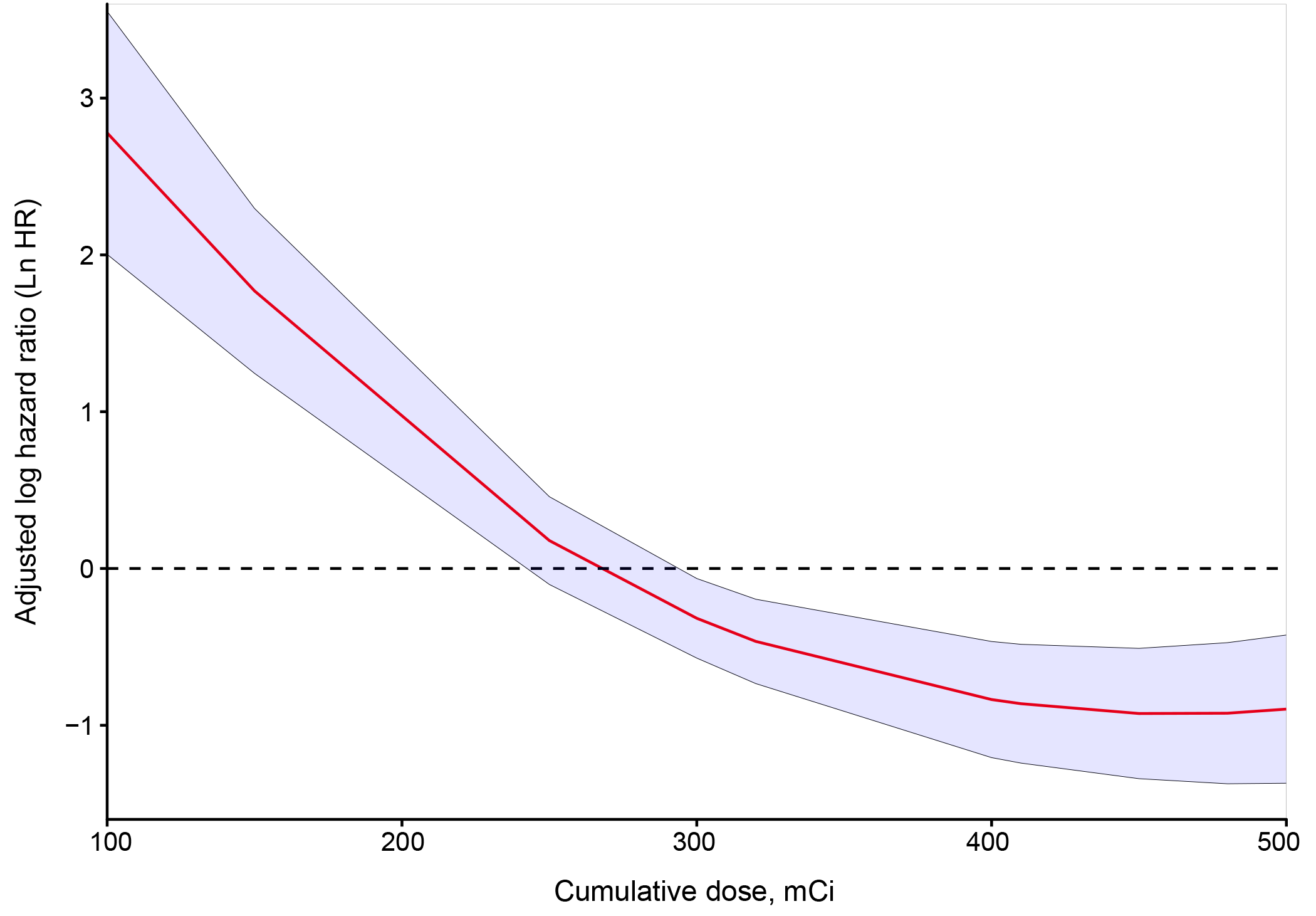


**eFigure 1.** Estimated logarithm hazard ratios (HRs) with 95% confidence intervals for the association of the cumulative dose of RAIT and OS in non-RAI-avid patients with DTC and PM.

*Adjusted by age, sex, pathology type, extrathyroidal extension, lymph-node involvement, extrapulmonary metastases, diameter of pulmonary metastases, and radiotherapy or chemotherapy.*


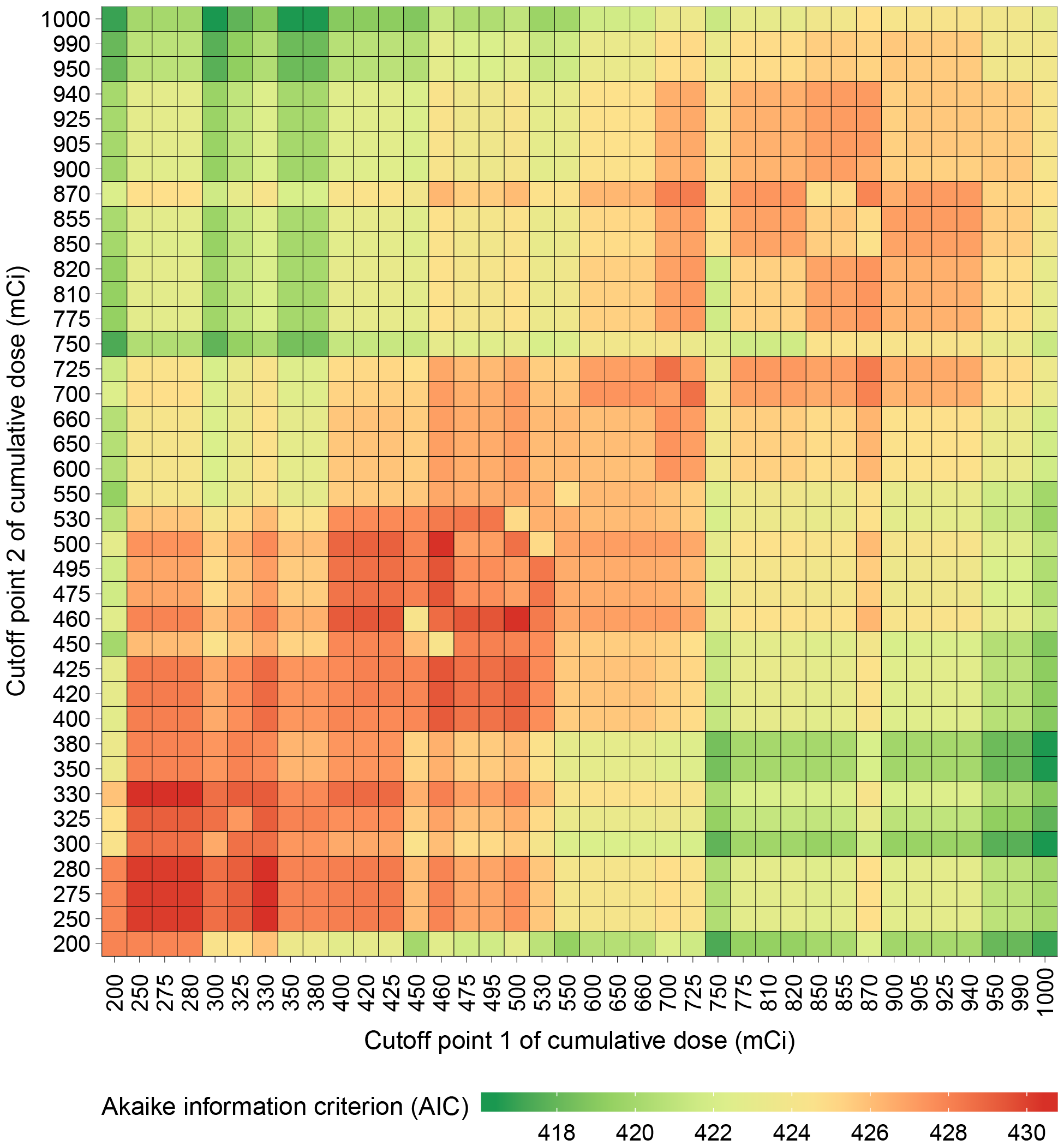


**eFigure 2.** Akaike Information Criterion (AIC) for models of association between binary and tertile cumulative doses of RAIT and OS.


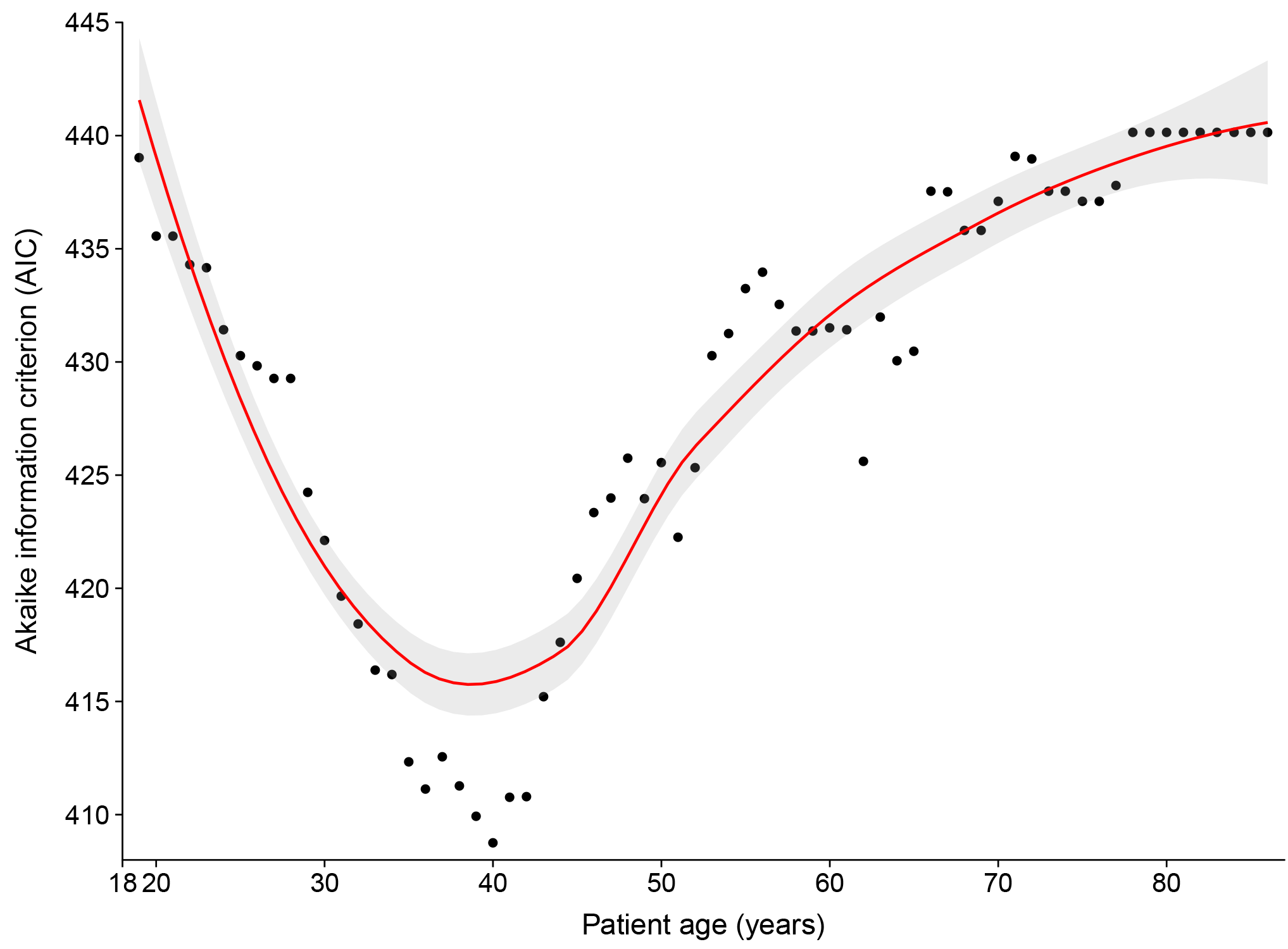


**eFigure 3.** AIC for models of association between age and OS.


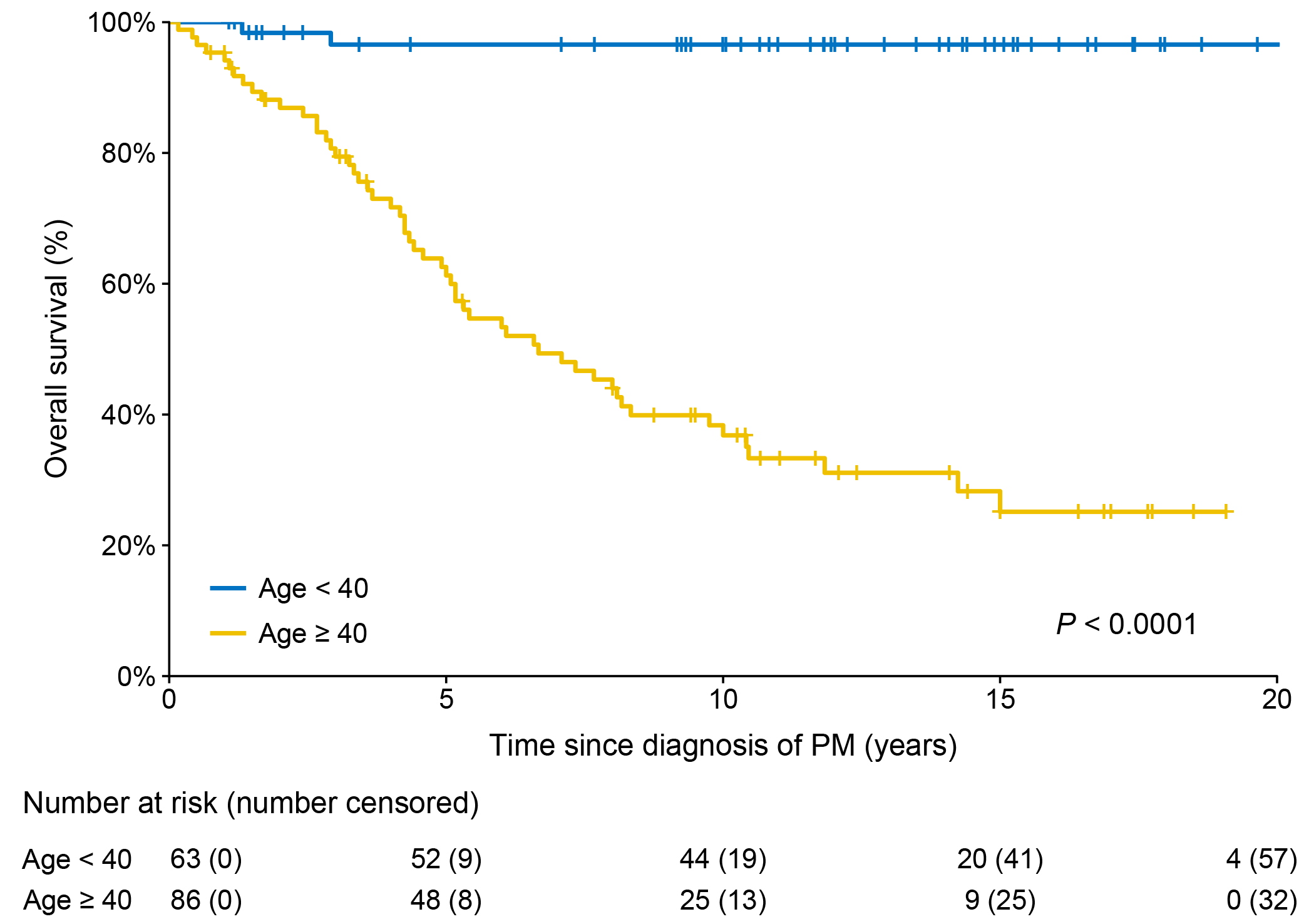


**eFigure 4.** Comparison of unadjusted Kaplan–Meier survival of age ≥ 40 years and < 40 years.
